# An assessment of primary care attributes from the perspective of female healthcare users^1^

**DOI:** 10.1590/0104-1169.0496.2587

**Published:** 2015-07-03

**Authors:** Eliane de Fátima Almeida Lima, Ana Inês Sousa, Cândida Caniçali Primo, Francielie Marabotti Costa Leite, Rita de Cassia Duarte Lima, Ethel Leonor Nóia Maciel

**Affiliations:** 2 PhD, Adjunct Professor, Departamento de Enfermagem, Universidade Federal do Espíríto Santo, Vitória, ES, Brazil; 3 PhD, Associate Professor, Escola de Enfermagem Anna Nery, Universidade Federal do Rio de Janeiro, Rio de Janeiro, RJ, Brazil; 4 Doctoral student, Escola de Enfermagem Anna Nery, Universidade Federal do Rio de Janeiro, Rio de Janeiro, RJ, Brazil. Assistant Professor, Departamento de Enfermagem, Universidade Federal do Espíríto Santo, Vitória, ES, Brazil; 5 Doctoral student, Departamento de Epidemiologia, Universidade Federal de Pelotas, Pelotas, RS, Brazil. Assistant Professor, Departamento de Enfermagem, Universidade Federal do Espíríto Santo, Vitória, ES, Brazil; 6 PhD, Associate Professor, Departamento de Enfermagem, Universidade Federal do Espíríto Santo, Vitória, ES, Brazil

**Keywords:** Primary Health Care, Family Healthcare, Healthcare Services Evaluation, Nursing

## Abstract

**OBJECTIVE:**

this study sought to assess the quality of the Family Health Strategy (FHS) and investigated the association between primary care attributes (PCAs) and the sociodemographic characteristics of users.

**METHOD:**

a total of 215 female FHS users were interviewed for this descriptive and cross-sectional study. The Primary Care Assessment Tool (PCATool), Adult Edition was used, and the results were analyzed using Fisher's exact tests, Pearson's chi-square tests and logistic regressions.

**RESULTS:**

the lowest average score corresponded to the dimension "accessibility" (1.80), and the highest score corresponded to "access" (8.76). The results corresponding to the attributes "longitudinality", "coordination", "comprehensiveness", and "orientation" were not significant. No association was found between the participants' sociodemographic characteristics and the essential, derivative, and general attributes (p>0.05).

**CONCLUSION:**

several attributes must be improved across all the investigated services from the perspective of female FHS users.

## Introduction

The Family Health Strategy (FHS) was established and implemented in Brazil to replace the traditional primary care model. The FHS has undergone massive expansion across the country's counties over the past decades. The organization of the new model at the municipal level follows preset operational guidelines that provide guidance regarding the functioning of units and staff practices, standardization per intervention area, and strategic lines of action and care delivery 1.

As the preferential path of access to the healthcare system, FHS represents the ideal locus to improve the management of care. Furthermore, the FHS implements comprehensive care, given its proximity to local users, elaborates particular therapeutic projects aimed at making healthcare more humane, gives better qualified and effective attention to users vis-à-vis both the spontaneous and organized demand, and provides comprehensive care to users^2^.

Because of its unique characteristics, FHS has been the target of various assessments with different foci. These analyses are relevant because they reflect different realities and therefore might contribute to strategies more narrowly centered on users' needs and the reformulation of staff work processes as well as the reorientation, prioritization, and improvement of managerial training vis-à-vis the new types of demands that have resulted from changes in social realities 3_._

Therefore, the inclusion of female users in the evaluation of the Unified Health System (Sistema Único de Saúde; SUS) enables researchers to gather different perceptions regarding the healthcare services because these women have distinct priorities that should be considered and reconsidered when assessing the quality of healthcare actions and services. The evaluation of the FHS from the perspective of users requires a systematic collection of information regarding activities, characteristics, and outcomes to make decisions aimed at improving efficacy, guiding future scheduling and team decision making, and increasing user satisfaction^4^.

As a function of the aforementioned considerations, the present study assessed the quality of the FHS from the perspective of female users and investigated the association between primary care attributes (PCAs) and these participants' sociodemographic characteristics. 

## Methods

The present cross-sectional, analytic, and quantitative study was conducted at healthcare units in Serra County, Espírito Santo, Brazil where the FHS was introduced more than 1 year earlier. The study participants were female users of the FHS, aged 20 to 49 years old. 

The following parameters were used to calculate the sample size: the population of women aged 20 to 49 years old enrolled in the FHS, equal to 32,540; a confidence level of 90%; a margin of error of 7%; and a proportion of 0.5 to maximize the sample size. The calculation was performed using EpiInfo. The random sample included 215 participants. 

The fieldwork team was composed of five female interviewers with no links to the investigated services. Four nurses and one nutritionist were duly trained to perform the interviews, and a private location was reserved to conduct them. The study coordinator, who was also the field supervisor, provided training. The questionnaire was pretested with 30 women within the same age range at municipal healthcare units. These women were not included in the actual study because the FHS was established less than 1 year earlier. The pretest evaluated the instrument and contributed to the interviewers' training and alignment; the results showed that no changes were needed.

The data were collected from August 5 to September 13, 2013. The interviews were conducted within the unit operating hours; no potential participant refused participation, and none was lost for analysis. The volunteers were selected from the group of women who met the inclusion criterion(i.e., a 20- to 49-year-oldFHS user) using a lottery method that resulted in a random sample. The selected women were invited to participate in the study and were informed of the study aims; their anonymity was ensured. Those who agreed to participate signed an informed consent form. 

The instrument used for data collection had two sections: The first was designed to investigate participants' sociodemographic profiles (age, marital status, educational level, occupation, economic status, number of children, and unit services used), and the second was the *Primary Care Assessment Tool *(PCATool),Adult Edition, which is composed of 87 items distributed across 10 components that correspond to the following PCAs: 1. Strength of affiliation with the healthcare service, three items; 2. First contact access: Use, three items; 3. First contact access: Accessibility, 12 items; 4. Longitudinality, 14 items; 5. Coordination: Integration of care, eight items; 6. Coordination: Information systems, three items; 7. Comprehensiveness: Services available, 22 items; 8. Comprehensiveness: Services received, 13 items; 9. Family centeredness, three items; and 10. Community orientation, six items. The items were responded to using a 4-point Likert scale (ranging from 1 to 4) with the following options: "definitely" (4), "probably" (3), "probably not" (2), "definitely not" (1) and "I don't know/don't remember" (9)^5^.

To assess the quality of the FHS, the average of the score for each item within each dimension and sub dimension was calculated as were the essential score (i.e., the average of the scores in the dimensions access, accessibility, longitudinality, integration of care, coordination, comprehensiveness, and strength of affiliation), derivative score (i.e., the average of the domains scores of family centeredness and community orientation), and total score (i.e., the average of the essential and derivative scores and the strength of affiliation). 

After the data were aggregated relative to each attribute, the values were transformed on a continuous scale ranging from 0 to 10 using the following equation: Scale = ([obtained score - 1]x10)/3. Scores ≥ 6.6 were considered high and equivalent to scores ≥ 3 on the Likert scale; scores ≤ 6.6 were considered low^5^.

The associations between PCAs and user characteristics were investigated using Pearson's chi-square tests, Fisher's exact tests and logistic regressions. The significance level was set to 5% with 95% confidence intervals. Data analysis was performed using STATA 12 and IBM SPSS Statistics, version19.

The research ethics committee of Anna Nery Nursing School, Rio de Janeiro approved this study(ruling no. 315,266).

## Results

A total of 215 interviews were conducted with female FHSusers in Serra County. The sociodemographic characteristics of the sample are described in Table 1. 

**Table 1.** Sociodemographic profile of FHS female users, Serra, ES, Brazil, 2013

Variables/categories		N	%
Marital status			
	Married	127	59.1
	Single	50	23.3
	Other	38	17.7
Children			
	Yes	191	88.8
	No	24	11.2
Educational level			
	Incomplete elementary school	64	29.7
	Complete elementary school	32	14.9
	Incomplete secondary school	24	11.1
	Complete secondary school	79	36.8
	Incomplete higher education	12	5.6
	Complete higher education	4	1.8
Economic class			
	A	1	0.5
	B	23	10.7
	C	154	71.6
	D	35	16.3
	E	2	0.9

Most of the participants were married (59.1%) and had children (88.8%). Approximately 36.8% of the sample had completed secondary school, whereas 29.7% had not completed elementary school. Most of the participants belonged to economic class C (71.6%). 

Table 2 describes the average scores that the participants attributed to the primary care dimensions.

**Table 2.** Scores attributed to the primary care dimensions by female FHS users, Serra, ES, Brazil, 2013

Dimensions	N	Minimum	Maximum	Mean	Standard deviation
Strength of affiliation	215	0.00	10.00	6.95	2.72
Access	215	3.33	10.00	8.76	1.67
Accessibility	215	0.00	6.11	1.80	1.45
Longitudinality	215	0.00	9.29	5.13	2.32
Coordination: Integration of care	92	1.00	4.00	2.77	0.74
Coordination: Information systems	215	0.00	10.00	5.68	2.49
Comprehensiveness: Services available	215	1.36	9.24	5.05	1.65
Comprehensiveness: Services received	215	0.51	12.05	3.94	2.16
Family centeredness	215	0.00	10.00	4.67	3.16
Community orientation	215	0.60	10.00	4.47	2.41

The dimension "accessibility" exhibited the lowest average score (1.80). The following additional attributes were given scores below the reference value (i.e., ≤ 6.6), exhibiting unsatisfactory results: "coordination: integration of care" (2.77), "comprehensiveness: services received" (3.94), and "community orientation" (4.47). 

The absolute score given to the attribute "coordination: integration of care" differed from all the others because it was only assessed when the participants had been referred to specialized care or services. 

The SDs of the attributes "family centeredness" (4.67; standard deviation [SD] = 3.16); "comprehensiveness: service received" (5.05; SD = 1.65); "coordination: information systems" (5.68; SD = 2.49); "longitudinality" (5.13; SD = 2.32); and "strength of affiliation" (6.95; SD = 2.72) were too close to the mean; therefore, they might modify the scores, resulting in satisfactory outcomes (score ≥ 6.6).In the case of the attribute "strength of affiliation", the mean might be reduced, resulting in an unsatisfactory outcome (score ≤ 6.6). Interestingly, the attribute with the highest score was "access" (8.76). 

The essential, derivative, and total scores were 5.35, 4.57, and 5.19, respectively, and thus low (≤ 6.6).

No associations were found between the participants' sociodemographic characteristics and the essential (strength of affiliation, accessibility, longitudinality, integration of care, coordination and comprehensiveness), derivative (family centeredness and community orientation), or total attributes (p >0.05). 

## Discussion

A previous study on the performance of primary care in São Paulo found that most service users were female and had completed elementary school^6^. Similarly, studies conducted at healthcare units in Ribeirão Preto, SP^7^ and São Luís, MA^8^ reported a higher prevalence of women, although most had not completed their elementary education^7^^-^ 8;thus, that sample was similar to the sample of the present study.

One study of 355 users performed at an urban center in southern Brazil found that most were female (67%), were married or in a stable union (57.2%), and had low family incomes 9; these data match those of the present study. Importantly, women with low educational levels and low incomes tend to marry or live with their partners earlier, start their sexual lives and have children sooner, and have more children^10^.Poor education might also lead to social exclusion because it denies the right to citizenship, thereby maintaining the cycle of poverty and marginality in addition to alienating the affected population from future opportunities 11.

The assessment of the primary care dimensions showed that strength of affiliation exhibited one of the highest scores, which suggests that the participants considered the FHS as a reference for healthcare. This finding corroborates the results of other studies conducted in Brazil^12^^-^ 13.

Participants gave the attribute "first contact access: use" the highest score, which shows that theysucceededin accessing municipal Family Health Units and were provided a modality of care. Studies performed in the interior of São Paulo and Minas Gerais corroborate this finding, rating access as adequate^12^^,^ 14. Both the opportunity to use healthcare services when needed and their geographical distribution comprise access 2.

A study conducted in Ribeirão Preto, SP, however, showed that 100% of female FHS users rated access as "very poor"^15^. Relative to the perceptions of caregivers assessed in a study performed in Montes Claros, the scores given to the attribute "first contact" were low^13^. 

In the present study, the attribute "accessibility" had the lowest score. These findings indicate a healthcare service problem because this attribute not only involves access to or the actual arrival of female users at the service but also several aspects related to receptivity and the humanization of care. Accessibility is understood as a balance in the supply and demand of services, and it includes features such as availability, comfort or delay in scheduling visits, inadequacy of schedules and the procedures for making appointments, free care, the waiting time for consultations, and other procedures^2^.

Users gave accessibility the highest score in a study performed in São Luís, MA (northeastern Brazil); units closing after 18:00 h and on weekends were described as the primary difficulty^8^.In addition, a study conducted in the Brazilian Midwest found that users were unsatisfied with the care available^16^, and difficulties in making appointments at specialized facilities and in obtaining access to medium- and high-complexity diagnostic and treatment services, long waiting times, long (actual or virtual) queues, and delays in the delivery of test results, among other problems, were reported in all the investigated northeastern counties in Brazil 17.

Unlike the present study, both professionals and users (approximately 50% of the total) assessed accessibility as good according to the maximum possible score for this dimension on the instrument^9^.

Receptivity plays a key role in the accessibility of users to health services because this is one of the major tools for the humanization of services and care provided. An adequate reception, effectiveness in problem solving, listening to users, meeting their needs, and comprehensive care are all essential elements of that process^10^.

Participants gave the attributes "coordination: integration of care", "comprehensiveness: services received", "family centeredness", and community orientation the lowest scores. These findings indicate the poor ability of the service to maintain longitudinality in the care provided and its comprehensiveness along the network of assistance.

Coordination of care consists of articulating the various services that comprise a healthcare network and the corresponding actions such that, regardless of the place where care is provided, care is synchronized and aims at a common goal (i.e., providing users the best possible assistance)^18^. Unlike the present study, one study conducted in Montes Claros, MG, showed that the evaluation of the attribute "coordination: integration of care" was close to the ideal regarding FHS teams, and no difference was found between the results of the assessment of FHS and other types of services, which indicated a fairly adequate level of coordination^13^. In addition, one study performed with users in São Paulo County, SP, found that the attribute coordination was considered satisfactory^19^. Another study found that mothers of infants younger than 1 year old rated the attribute "coordination of services" as adequate^12^;in another study, the participants rated that attribute as unsatisfactory^20^. A study performed in Ribeirão Preto, SP found that 75% of users rated "coordination of services" as poor 15. 

Importantly, several factors influence the coordination of services within the primary care setting, among which the following stand out: increasing the participation of general practitioners regarding the management and responsibility of users' treatments through the healthcare network; increasing the problem-solving abilities of primary care healthcare workers through the allotment of resources and the enlargement of the scope of the services provided; and creating well-established referrals and counter-referrals along the therapeutic route 21.

Similar to the attribute "coordination of services", the participants also rated "comprehensiveness" as poor. This finding matches the results of other studies performed in Brazil^12^^,^ 22 ^-^ 23. Although comprehensiveness involves coordination and cooperation among healthcare providers to develop a genuine healthcare system, this is not the case in the daily practice of SUS services. To solve this problem, the healthcare networks are being strengthened to ensure the integration of services and the coordination of the care provided to users along their therapeutic process 24.

Comprehensiveness is present when activities related to the satisfaction of the population's needs are performed. These activities include vaccination, performing indicated procedures and tests, and detecting and addressing community health problems^2^.

Unlike the present study, the attributes "comprehensiveness" (health promotion and receiving preventive actions) and "longitudinality" were rated as satisfactory and given high scores (≥ 6.6) by the users of the services in Montes Claros, MG^13^."Longitudinality" was also a strong point according to the assessment of primary care from the perspective of older adults^23^ and the only attribute considered satisfactory by the caregivers of children under 2 years old^22^.

In addition, the participants rated the attributes "family centeredness" and "community orientation" as poor in the present study, which is similar to the results reported in other studies in which the users rated those attributes as unsatisfactory^6^^,^ 25. 

Therefore, the attribute "community orientation", which aims to ensure the health of each individual user in the area and the community as a whole, suggests that professionals should act directly by performing social mobilization and participating in its improvement. Thus, professionals must have accurate knowledge of the community, identify its health problems, and develop and adjust healthcare actions to respond to such problems and monitor the effectiveness of the actions^2^.

Importantly, a lack of commitment, respect, adequate receptivity, accessibility, singular therapeutic projects, coordination, and comprehensiveness of services are the main causes of user dissatisfaction. Moreover, the quality of care and user satisfaction are directly related to access to healthcare services from a perspective of receptive approach that ensures the continuity and coordination of care. Therefore, the user perception of the FHS is of paramount importance because it provides the opportunity to verify the community's response to the supply of services and to adjust the latter to the former's expectations^3^.

## Conclusions

The results of the present study are not only relevant to the investigated services but also to the healthcare public policies of Serra County. According to the users, some attributes must be improved in all the services assessed.

We conclude that the dimension "accessibility" exhibited the lowest average score (1.80). The results relative to the attributes "longitudinality", "coordination", "comprehensiveness" and "orientation" were also unsatisfactory. Conversely, the dimension "access" was rated highest by the FHS users (8.76). Together, these results indicate that even though users are able to access these services, this access does not ensure that they will receive high-quality and timely care. 

The assessment of user care at public healthcare services might allow healthcare workers to express their opinions and perceptions regarding the quality of the available services as well as strengthen their participation in the processes of planning and social control. This development would enable interventions better adjusted to the problems encountered in the daily operation of services, providing advances in care and the management of healthcare and nursing services. 

Although the female users of the FHS in Serra, ES had low educational levels and belonged to the lowest social classes, they exhibited a critical attitudes vis-à-vis the FHS and its available services. In this regard, the results of the present study conflict with previous researchin which the users with lower family incomes and cultural and educational levels tended to assess the healthcare services in a more positive light and were more satisfied with the available services. The results of the present study indicate a positive aspect with regard to the participants' empowerment through social and citizen strengthening given that they showed active and critical attitudes. 

Regarding the limitations of the present study, its results correspond to a single county. In addition, this study was a cross-sectional evaluation; thus, it is subject to the limitations inherent to this type of design. Nevertheless, the lack of studies in Brazil on the subject addressed here indicates the relevance of similar assessments.
